# Disentangling the latent space of GANs for semantic face editing

**DOI:** 10.1371/journal.pone.0293496

**Published:** 2023-10-26

**Authors:** Yongjie Niu, Mingquan Zhou, Zhan Li

**Affiliations:** 1 School of Information Science and Technology, Northwest University, Xi’an, China; 2 College of Mathematics and Computer Science, Yan’an University, Yan’an, China; Nanchang University, CHINA

## Abstract

Disentanglement research is a critical and important issue in the field of image editing. In order to perform disentangled editing on images generated by generative models, this paper presents an unsupervised, model-agnostic, two-stage trained editing framework. This work addresses the problem of discovering interpretable, disentangled directions of edited image attributes in the latent space of generative models. This effort’s primary objective was to address the limitations discovered in previous research, mainly (a) the discovered editing directions are interpretable but significantly entangled, i.e., changes to one attribute affect the others and (b) Prior research has utilized direction discovery and direction disentanglement separately, and they can’t work synergistically. More specifically, this paper proposes a two-stage training method that discovers the editing direction with semantics, perturbs the dimension of the direction vector, adjusts it with a penalty mechanism, and makes the editing direction more disentangled. This allows easy distinguishable image editing, such as age and facial expressions in facial images. Experimentally compared to other methods, the proposed method outperforms them both qualitatively and quantitatively in terms of interpretability, disentanglement, and distinguishability of the generated images. The implementation of our method is available at https://github.com/ydniuyongjie/twoStageForFaceEdit.

## Introduction

Generative Adversarial Networks [1] (GANs) have emerged as the dominant generative learning paradigm, showing clear superiority in the quality of generating realistic, diverse images [2–6]. BigGAN [2] and StyleGAN2 [5] are actually the best models in terms of realism, variety, and clarity of image generation, and a lot of research has considered both models. However, the GAN model does not provide an inherent way to understand and manipulate these generating factors. In fact, the GAN model functions are emphasized as a "black box" in many applications. Thus, researchers are investigating the GAN latent space’s structure to develop a method that can discover interpretable and disentangled attribute editing directions.

Methods for discovering the direction of attribute editing in the latent space of GANs are divided into supervised and non-supervised branches. The supervised methods are able to discover editing directions in the latent space that are consistent with supervisory control factors [4,7,8]. Supervision is in the form of labels assigned to the generated images, either by explicit human annotation or by the use of pretrained semantic classifiers such as linear support vector machines. More recent works [9–11] manipulate editing directions in a well-aligned latent space with some controllable manipulations (e.g., zoom, translation) in image space. Supervised methods are limited by many factors, such as the assumption that the latent space has editing operations specified by supervised conditions, the accuracy of human annotation, the fact that the number of editing directions is less than or equal to the number of semantic classifiers, etc.

Another research direction for discovering editing directions in the latent space of generative models is unsupervised methods. The SeFa [12] method decomposes the weights of the first layer of the StyleGAN into feature vectors, which are the editing directions, and then identifies the specific semantics of the directions in a post-processing way. GANSpace [13] performs PCA on the intermediate vector matrix, formed by a large number of samples, in the latent space of the generative model. Thus, it obtains a set of non-orthogonal editing directions and performs hierarchical editing on the generator to achieve a certain degree of disentangled attribute editing. Like other methods, the method is a very demanding training process that requires sampling a large number of random latent codes and regressing latent directions. Added to that, Voynov *et al*. [14] proposed an unsupervised method to discover editing directions in the latent space by relying on the classification loss of the classification network, which theoretically can discover almost all interesting directions in the latent space. To sum up, the evaluation of the above methods basically relies on subjective visual inspection or laborious human labeling.

Learning disentangled representations generates several advantages for many computer vision tasks, such as controllable image generation [12,15,16], image manipulation [17], and adversarial attacks [18,19]. In recent years, unsupervised disentanglement learning has attracted substantial attention. Moreover, many disentanglement methods [12,15,16,20–22] have been proposed. Zhu *et al*. [16] encouraged GANs to learn disentangled representations during training through Variation Predictability (VP) loss and proposed a VP metric to quantitatively evaluate disentanglement. Added to that, the Hessian Penalty [15] encourages learning a disentangled representation by minimizing the off-diagonal entries of the output’s Hessian matrix with respect to its input. Finally, OroJaR [23], introduced by Wei *et al*., is basically the same as the Hessian Penalty principle. **To sum up, most of the research nowadays belongs to the integrative method, that is, the relevant disentanglement mechanism is integrated into the model training process.**

Inspired by Voynov’s work [14] and the Hessian Penalty [15], we propose an unsupervised, model-agnostic, two-stage training framework for disentangled editing. The two stages are:

- Direction discovery: given a trained generative model G and using the classification and regression losses of a classification network, discover all directions in the latent space with editing capabilities;
- Disentanglement learning: The dimension of the discovered editing direction is subjected to a minor perturbation, and the sum of squares of the off-diagonal elements of the Hessian matrix between the perturbation-generated image and the perturbation is minimized to accomplish disentanglement of the editing direction.

**Fig 1:**
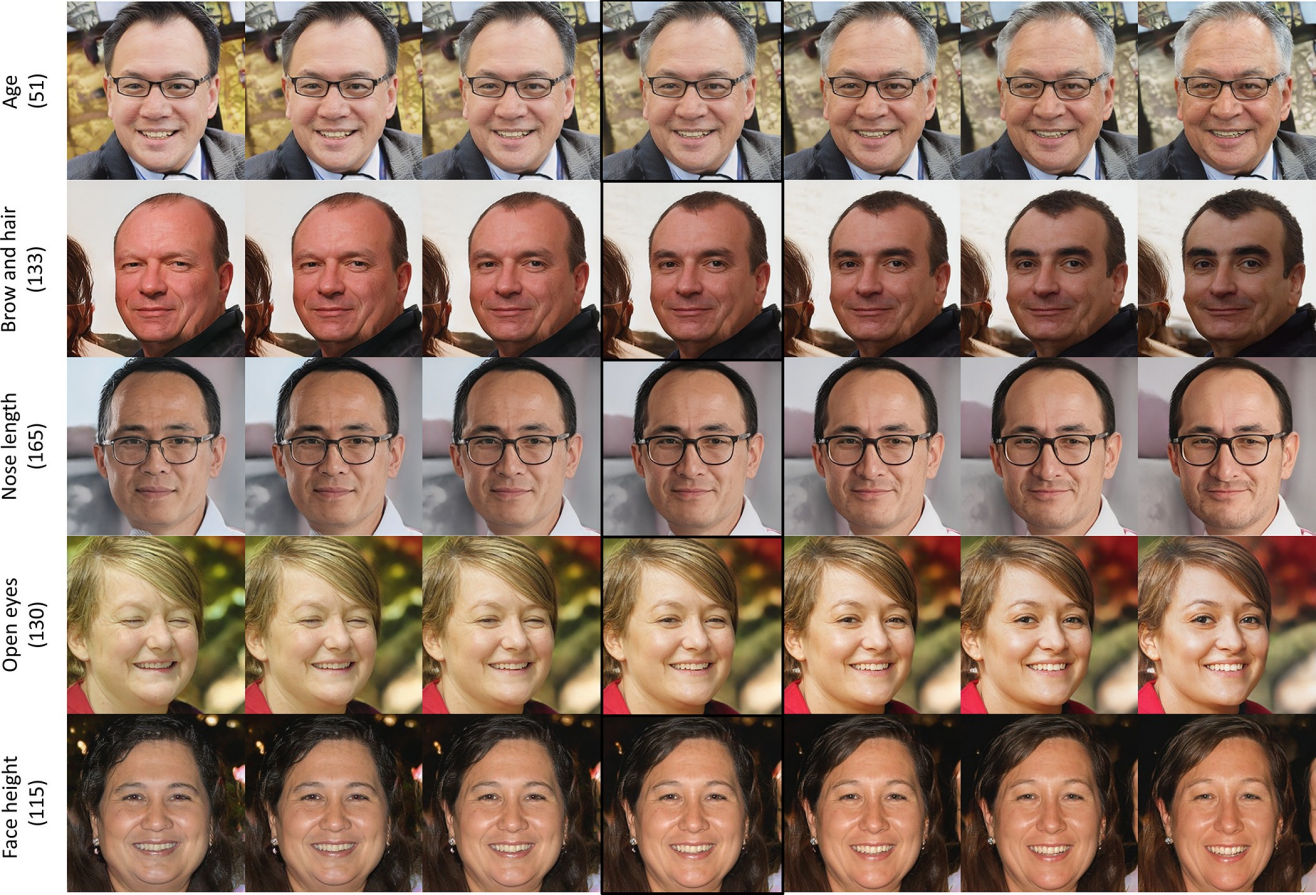
Examples of disentangled editing directions discovered by our method.

Fig 1 shows an example of exciting and disentangling obtained using the proposed method. The images with borders in Fig 1 are the original images. The left side of this original image represents the decreasing negative editing direction, and the right side represents the gradually increasing positive direction. As can be seen from Fig 1, our method can disentangle other image attributes while modifying some attributes.

Inspired by SeFa [12] and InterFaceGAN [7], we propose some methods to quantitatively evaluate disentanglement and to automatically identify the semantics of the discovery directions. One of the methods consists of traversing the discovered editing directions, generating image sequences, using different trained kinds of attribute classifiers to score different attributes of the image sequence, and calculating the average Pearson’s correlation between attribute scores and traversal indices. Arrange the correlation coefficients of each attribute in all directions in descending order, and each attribute is the semantics of the direction with the largest correlation coefficient, which can automatically determine the correspondence between image attributes and editing directions without manual identification. The contrast between different attribute correlation coefficients in one editing direction can quantitatively show the degree of disentanglement in that direction. Based on the realized experimental simulations, the directions discovered after the two-stage training are more interpretable and disentangled than other methods. It is found that the semantics of the automatically determined editing direction are largely consistent with the manual annotation.

To sum up, the main contributions of this paper can be summarized as follows:

- We propose an unsupervised, model-agnostic, two-stage training strategy. The method discovers all editing directions in the latent space of a pre-trained generative model in the first stage and then employs a penalty mechanism to disentangle the discovered directions in the second stage. Thus, disentanglement of discovery directions is improved, i.e., modifying an image attribute corresponding to semantics in one direction will have as minimal impact on other attributes as possible;
- We propose methods to automatically determine the semantics of directions and to quantitatively evaluate the performance of the directional disentanglement. Using the pre-trained image attribute classifier to score various attributes of the generated image sequence, calculating the average Pearson’s correlation between the attribute score and the image editing path, and determining the semantics of the direction by sorting the result of the attribute correlation coefficient are the main steps that are required to apply these methods. The comparison of the correlation coefficients of different attributes in an editing direction can quantitatively evaluate the degree of disentanglement in this direction;
- Using the new method on the generative model StyleGAN2 and comparing it with other methods both qualitatively and quantitatively, it is found that our method can not only discover meaningful semantic directions but also have better disentanglement performance.

Finally, this paper is divided as follows: In the next section, several related works to the proposed issue are presented and analyzed, whereas Section 3 shows the methodology of work applied in this paper. In Section 4, the experimental details, results, and some discussion and analysis are presented, and Section 5 summarizes the obtained conclusions and recommends some future research directions.

## Related work

### Discovering editing directions in the latent space

The discovery of editing directions in the latent space has two branches: the supervised method and the unsupervised method. Radford *et al*. [24] were the first to discover the phenomenon that latent codes exhibit the features of arithmetic operations. By modifying these latent codes, it is possible to change several expressions and add certain accessories to the face. Due to this discovery, image editing became much easier and got extensive research attention. InterFaceGAN [4] uses the ResNet-50 network [25] to train an auxiliary attribute prediction model based on the CelelbA dataset [26] to predict attributes for the sampled 500 thousand generated images. Five linear SVMs were trained using a set of data pairs that included attribute scores and latent encoding. The hyperplane of each SVM is the editing direction of the associated attribute. As a result, InterFaceGAN can only determine the editing direction of five binary attributes and necessitates the use of an auxiliary attribute prediction model and SVM training. StyleSpace [27], another technique relying on human supervision, uses the pre-trained image segmentation network to identify and edit local semantic regions in the style channel; thus, accurate editing for the local face regions is possible; however, it is difficult to generalize this method to other generative models because it goes deep into the interior of a specific model. Plumerault *et al*. [11] proposed an algorithm that translates the image *Is*, generated by the latent code *z*, into another image *Ir* through the transformation *T*_*δ*_ of the intensity *δ*, where *I2* obtained the latent code *z*_*t*_ through the GAN inversion. Based on the basic hypothesis that the parameter *δ* of a specific factor of variation can be predicted from the coordinate of the latent code along an axis *μ*, the direction *μ* of the transformation *T*_*δ*_ in the latent space is solved by identifying the values of the (*z*, *z*_*t*_, *δ*). The method focuses on image editing mostly limited to domain-agnostic factors (e.g., zoom scale or translation).

SeFa [12] finds the most important eigenvectors by decomposing the weights of the pre-trained generative model’s first layer and sorting them by eigenvalues. The eigenvectors vectors represent editing directions, and the specific semantics of these directions will be recognized by humans afterwards. GANSpace [13] samples a lot (10^6^) to obtain the corresponding intermediate latent *W* matrix. The basis obtained by performing PCA on the matrix *W* is the searched editing directions, which have rich semantic information. Eliezer *et al*. [9] obtained a closed-form expression corresponding to the transformation of the weights *W* and *b* of the first layer without applying any type of training or optimization. Voynov *et al*. [14] put the discovered editing directions into an external matrix via reconstructor classification and regression losses. The model-agnostic characteristics of this method make it applicable in many domains [15,23,28], even though they evaluate the performance of their method using human assessors’ judgments. Finally, Wang *et al*. [29] integrated these approaches by treating them as special cases of computing the spectrum of the Hessian for the LPIPS [30] model with respect to the input.

Supervised methods often require costly human labeling or training of specific auxiliary networks, vast quantities of sampling, and a limited number of directions to discover. Unsupervised methods can discover practically all editing directions; however, the identified directions are typically entangled with each other.

### Disentanglement learning

StyleSpace [27] provides a comprehensive description of entanglement learning where each latent dimension controls only a single visual attribute (disentanglement), and each attribute is controlled by only a single dimension (completeness). Two kinds of methods, i.e., the post-processing and the integration methods, have been mainly investigated for finding disentangled representations in GAN.

The integration method incorporates disentanglement learning into the model training process in such a way that the generative model has inherent disentanglement characteristics. InfoGAN [31] achieved the disentangled representations by maximizing the mutual information between the input latent variables and the output of the generator. Zhu *et al*. [16] presented a variation predictability loss that encourages disentanglement by maximizing the mutual information between latent variations and their corresponding image pairs. In addition, GAN-Control [32] encodes attribute information into sub-spaces of latent encoding *z* and trains the GAN model using factorized contrastive loss defined by the contrastive learning. In the process of training a new model from scratch, the Hessian Penalty is able to disentangle several crucial generative factors. To sum up, these studies provide comprehensive theoretical insights; In contrast to state-of-the-art GANs, however, they are typically applied to experimental or low-resolution datasets and generate inferior results in terms of quality and diversity.

The post-processing techniques are used to locate and identify interpretable directions in the pre-trained GAN’s latent space. Currently, the research community pays very little attention to post-processing methods, and many researchers tend to treat disentanglement learning as an incidental characteristic of the direction discovery. Hessian Penalty and Orthogonal Jacobian Regularization [15,23] were used as direction discovery tools on the BigGAN model, and a small number of editing directions, including rotation, scaling, and color transformation, were found. However, **our work uses the Hessian Penalty as a proprietary tool for disentanglement rather than direction discovery**.

### Proposed method

**Fig 2:**
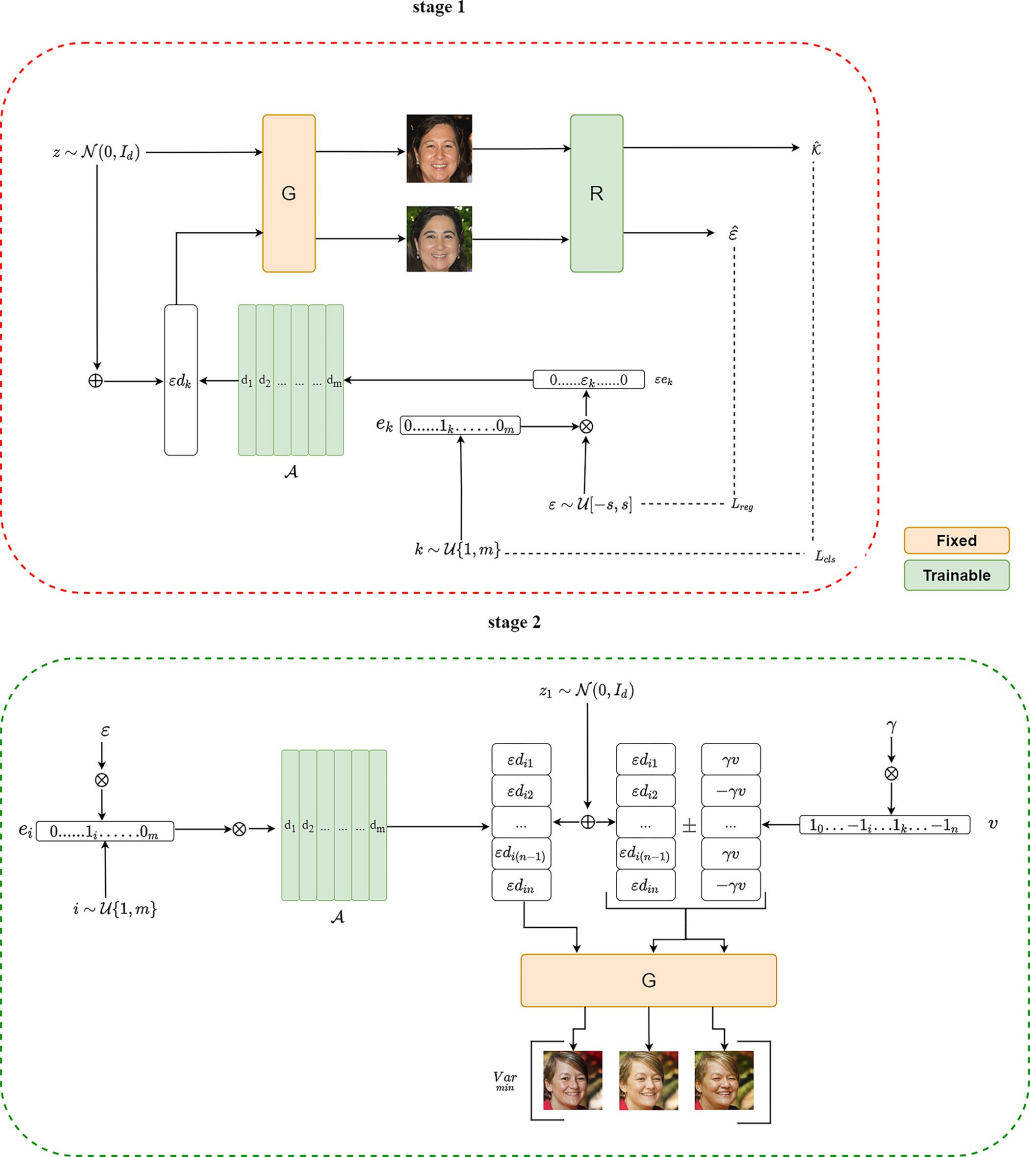
Overview of the proposed method.

An overview of our proposed method is shown in Fig 2.

Our primary objective is to develop strategies that simultaneously discover editing directions and disentangle them in the latent space of generative models. After theoretical investigation and experimental verification, a two-stage training method was proposed. The first stage consists of discovering semantically meaningful editing directions in the latent space and storing them in an external matrix *A*, and the second stage uses a Hessian Penalty to locally adjust the discovered editing directions to disentangle the directions from each other. The major focus of the two-stage training manipulation is the matrix *A* that is placed outside the generative model. Hence, the proposed method is model-agnostic and can be easily adapted for usage in different generative models.

After completing model training, the columns of matrix *A* represent the disentanglement editing directions. In order to verify the effectiveness of our method, images are generated after random sampling in the latent space of the pre-trained StyleGAN2 model, and the generated images are edited using the editing direction in matrix *A*. **So all images in this paper are completely synthesized from the beginning. The images and figures in this article have been authorized by all authors to be freely available without restriction.**

### Discovering editing directions

The main task of the first stage is to discover meaningful editing directions in the latent space, which correspond to the dashed box on the top in Fig 2.

Randomly sampling a latent code *z ∼ N (0*, *I*_*d*_*)* from the latent space where *|z|* denotes the dimensionality of the vector *z*. For the StyleGAN2 model, *|z|* = n = 512.Input *z* into the generative model *G* to obtain the original image *I*_*origin*_ *= G(z)*. Then, a direction index *k* is generated in the interval *{1*,…, *m}* using a discrete uniform distribution where *m* is the number of directions expected to be discovered, and one-hot encoding, with *k* serving as an index, yields the direction extraction vector *e*_*k*_ *= (0*,…, *1*_*k*_, …, *0)*. After that, a random shift magnitude *ε* is generated over a continuous uniform distribution [*−s*, *s*] where *e*_*k*_*Aε* extracts the *k*th column from the matrix *A* as a candidate direction, and each column of the matrix *A∈R*^*|z|×m*^ represents a direction vector. Hence, *G(z + e*_*k*_*Aε)* generates the altered image *I*_*alter*_. The two images are connected on the channel and fed to the reconstructor R composed by ResNet-18, which produces two outputs $(\hat{k},\hat{\varepsilon }).\hat{k}$ is a prediction of direction index *k* and $\hat{\varepsilon }$ is a prediction of shift magnitude *ε*. Adjusting *A* and *R* by minimizing the loss function leads to have Eq (1).


$$\underset{A,R}{min}\;\underset{z,k,\varepsilon }{E}L(A,R)=\underset{A,R}{min}\;\underset{z,k,\varepsilon }{E}[L_{cls}(k,\hat{k})+\lambda L_{reg}(\varepsilon ,\hat{\varepsilon })]$$ (1)

For the classification term *L*_*cls*_*(·*, *·)*, the cross-entropy function is used, and for the regression term *L*_*reg*_*(·*, *·)*, the mean absolute error is used. In all experiments, we use a weight coefficient *λ* = 0.25, which is taken from Voynov’s experimental values.

### Directional disentanglement learning

The main task of the second stage is to disentangle the discovered direction vectors, which correspond to the dashed box on the bottom of Fig 2. After the first stage of training, each column in matrix *A* represents an editing direction. The optimal direction of disentanglement is to select one of the columns; when moving in this direction, only one attribute of the generated image is modified, while the others remain unchanged.

To simplify the problem, consider one dimension of the image *I*_*alter*_ first. The function *F*: ℝ^|*z*|^→ℝ is a scalar function that maps *z* to a dimension of the image *I*_*alter*_. Let any off-diagonal element *H*_*ij*_ of the Hessian matrix H of the function *F* with respect to *z* be 0, as given in Eq (2).


$$H_{ij}=\frac{\partial^{2}F}{\partial z_{i}\partial z_{j}}=\frac{\partial }{\partial z_{j}}(\frac{\partial F}{\partial z_{i}})=0,i\neq j$$ (2)

Where the inner derivative with respect to *z*_*i*_ represents the effect of a perturbation on the output of the function *F*, whereas, if the outer derivative with respect to *z*_*j*_ of the inner derivative is zero, it means that $\frac{\partial F}{\partial z_{i}}$ is independent of *z*_*j*_. In other words, as we change *z*_*i*_, *z*_*j*_ has no effect on how the function *F* output changes and vice versa.

Summing the squares of all non-diagonal elements of the *H* matrix, one can write:

$$L_{H}(F)=\sum_{i=1}^{\left|z\right|}\;\sum_{j\neq i}^{\left|z\right|}\;H_{ij}^{2}$$ (3)

For the whole image *I*_*alter*_ has *w × h* dimensions, and all ℒ_*H*_(*F*) functions form a set. For the generator G, take the maximum in the set to get the function:

$$L_{H}(G)=\underset{i}{max}L_{H_{i}}(F)$$ (4)

Minimizing the function of Eq (4) yields to the overall Hessian Penalty, which requires each non-diagonal element to be minimum, allowing each dimension of the output image to be disentangled relatively to each dimension of the input; Thus, one can conclude that the Hessian Penalty has a stronger disentangle capability than the OroJaR method, which controls the Jacobian matrix in a holistic manner.

However, it is impractical and slow to compute the Hessian matrices in Eq (4) during training when the dimensionality of the generated image is large. Thus, we can express Eq (4) in a different manner that admits unbiased stochastic approximations as represented in Eq (5) [15].


$$L_{H}(G)={Var}_{v}(v^{T}Hv)$$ (5)

Where *v* are Rademacher vectors (each entry has equal probability of being −1or +1).

In order to quickly compute *v*^*T*^*Hv* in Eq 5, we can do this via a second-order central finite difference approximation:

$$v^{T}Hv\approx \frac{1}{\gamma^{2}}[G(z+\gamma v)-2G(z)+G(z-\gamma v)]$$ (6)

Directly optimizing $A^{*}=arg\;\underset{A}{min}\;E_{z,e_{k},\varepsilon }L_{H}(G(z+\varepsilon Ae_{k}))$, where Hessian Penalty is now achieved using *e*_*k*_ instead of *z*; thus, Eq (6) will allow to write Eq (7).


$$v^{T}Hv\approx \frac{1}{\gamma^{2}}[G(z+(\varepsilon Ae_{k}+\gamma v))-2G(z+\varepsilon Ae_{k})+G(z+({\varepsilon Ae}_{k}-\gamma v))]$$ (7)

Eq (7) implies a minor perturbation *γv* on the *k*-th column (*Ae*_*k*_) of the orientation matrix such that the variance between the perturbed image and the original image is minimized and the editing direction in the matrix *A* is adjusted in such a way that the direction is disentangled. The variables γ and *ε* are hyperparameters to adjust the intensity of the perturbation, which in practice is γ = 0.1 and *ε* = 1.

Base on the StyleGAN2 model, as the *W* space has superior disentanglement qualities than the *Z* space, the above training is also applicable to the *W* space, and the output effect is better visualized than that on the *Z* space. Thus, our experiments are carried out in the *W* space.

### Advantages of two-stages over one stage

Each column in matrix *A* represents an editing direction, and the loss function of the first stage training does not explicitly restrict the direction vectors to being different from each other. To prevent the model from collapsing quickly into a small number of directions, i.e., the number of valid editing directions finally obtained is much smaller than the number found by the plan, and the remaining directions are parallel to the resultant ones; thus, the column vectors of matrix *A* are unit-normed and orthogonal by applying Gram-Schmidt processing [15] after each iteration. The orthogonalization prevents the collapse of the method, but it makes the final editing direction less precise. So far, there is no relevant evidence to verify that the editing direction must be necessarily orthogonal. For instance, the editing direction found by GANSpace is non-orthogonal. If a Hessian Penalty with a weak prior is also used in the first stage, the orthogonal operation will completely cancel out its fine-tuning of the editing direction. This is one reason for adopting the two-stage training protocol.

In more details, the first stage extracts the directions in matrix *A* by indexing *e*_*k*_, treating the columns in matrix *A* as a whole, and the granularity of the operation is to compute the vector. The Hessian penalty is effectively obtained through the perturbation of each dimension in the direction vector; thus, the dimension is the granularity of the Hessian Penalty operation. Since the two operations are at different levels, they are performed in two different stages. If one-stage is used, the editing direction in matrix *A* is basically a random vector without any semantics at the beginning of the algorithm and adjusting it with the Hessian Penalty to modify the editing direction is not in accordance with the idea of Hessian Penalty. Added to that, it will create a conflict with the direction discovery, which will make the classification prediction less accurate and the convergence slower. In the next section, experimental results will verify our idea.

## Experiments

In this section, we start to implement the proposed method and compare it with other methods using both qualitative and quantitative approaches.

The pre-trained model StyleGAN2 with a resolution of 256×256 can be obtained from the GitHub website (https://github.com/rosinality/stylegan2-pytorch). **All images in the experiments are generated by this model, so all images are available without restriction.**

### Implementation details

The experiments were carried out on a single V100 32G card from the V-Series, and the generation model employed the StyleGAN2 model trained on FFHQ [5], which is currently state-of-the-art in the field of face generation. To increase the size of the batch during training, the resolution of the generated images is set to 256×256 pixels. Five distinct types of experiments were developed to test our method. They are described here below:

1. Voynov’s method is not implemented on StyleGAN2, which is reimplemented and symbolized by the abbreviation SDD (Single Discovery Directions);
2. Peebles *et al*. [15] employ the Hessian Penalty as direction discovery on BigGAN and reimplement it on StyleGAN2 using SHP (Single Hessian Penalty) notation;
3. Integrating Voynov’s technique with Hessian Penalty together in one training stage is called HDP (Hybrid Discovery and Penalty). Due to the addition of the Hessian Penalty constraint to the HDP, the complete loss function of the model is shown in Eq (8):
$$\underset{A,R}{min}\;\underset{z,k,\varepsilon }{E}L(A,R)=\underset{A,R}{min}\;\underset{z,k,\varepsilon }{E}[L_{cl}(k,\hat{k})+\lambda L_{re}(\varepsilon ,\hat{\varepsilon })+\gamma L_{H}(G)]$$ (8)
  Where γ = 0.5, *λ* = 0.25;
4. In order to compare Hessian Penalty and OroJaR in terms of disentanglement learning, a method using OroJaR was implemented in the second stage, denoted by DTJ (Discovery To OroJaR);
5. Our method utilizes the DTH (Discovery to Hessian Penalty) notation.

The range of the shift variable magnitude *ε* is set to [–6, 6] for all methods, and the directional discovery number *m* is set to 256. The perturbation variable magnitude *γ* for the Hessian Penalty and OroJaR methods is set to 0.1. The number of iterations for the three methods of SHP, HDP, and HDP is equal to 10^5^. The first stage of both DTH, DTJ methods employs the model trained by SDD method where the second stage iteration number is also equal to 10^5^. The batch size for SDD is 32, 18 for all three SHP, DTH, and DTJ methods, and 12 for HDP.

Methods SDD, SHP, and HDP all initialize the matrix *A* with the standard normal distribution, and each iteration of the algorithm orthogonalizes the columns of the matrix. In the first stage of DTH and DTJ training, the initialization and operation of matrix *A* are identical to the method described above, and the second stage of training is initialized with the matrix *A* obtained from the first stage.

To quantitatively evaluate the preceding methods, the editing direction was traversed first: Using a fixed latent code z, a sequence of images is generated by traversing a fixed step in the positive and negative directions along a particular edit direction, producing one image per step; Subsequently, various pretrained classification networks are applied to score multiple attributes of each image in the image sequence.

The proposed attributes and pretrained classification networks are listed here below:

- the width and height of the face, using [33];
- the age, race, and gender score using FairFace [34];
- an identity score for each image of the sequence that expresses the similarity between the original image (central image of the sequence) and each of the remaining images, using ArcFace [35];
- More expressions for the face, e.g., au_1_Inner_Brow_Raiser, au_9_Nose_Wrinkler, au_12_Lip_Corner_Puller, etc., using DISFA [36,37].

Each image sequence corresponds to an editing direction. Calculate the Pearson correlation coefficient between the sequence score and sequence index for each attribute; as the evaluation standard, the mean value of the corresponding Pearson correlation coefficient was calculated after 200 distinct samples. then, the magnitude of the correlation coefficient of each attribute in the editing direction is compared to evaluate the disentanglement performance of that editing direction.

### Qualitative evaluation of face editing

By displaying the effects of different ways on face editing, the comparison demonstrates that our method is better than other methods in terms of disentanglement. Each method discovers a collection of editing directions, and for each editing direction, the traversal produces an image sequence. Thus, the visualization of this sequence illustrates the change in image attributes. For fair comparison, all methods use the same step length and the same number of steps.

**Fig 3:**
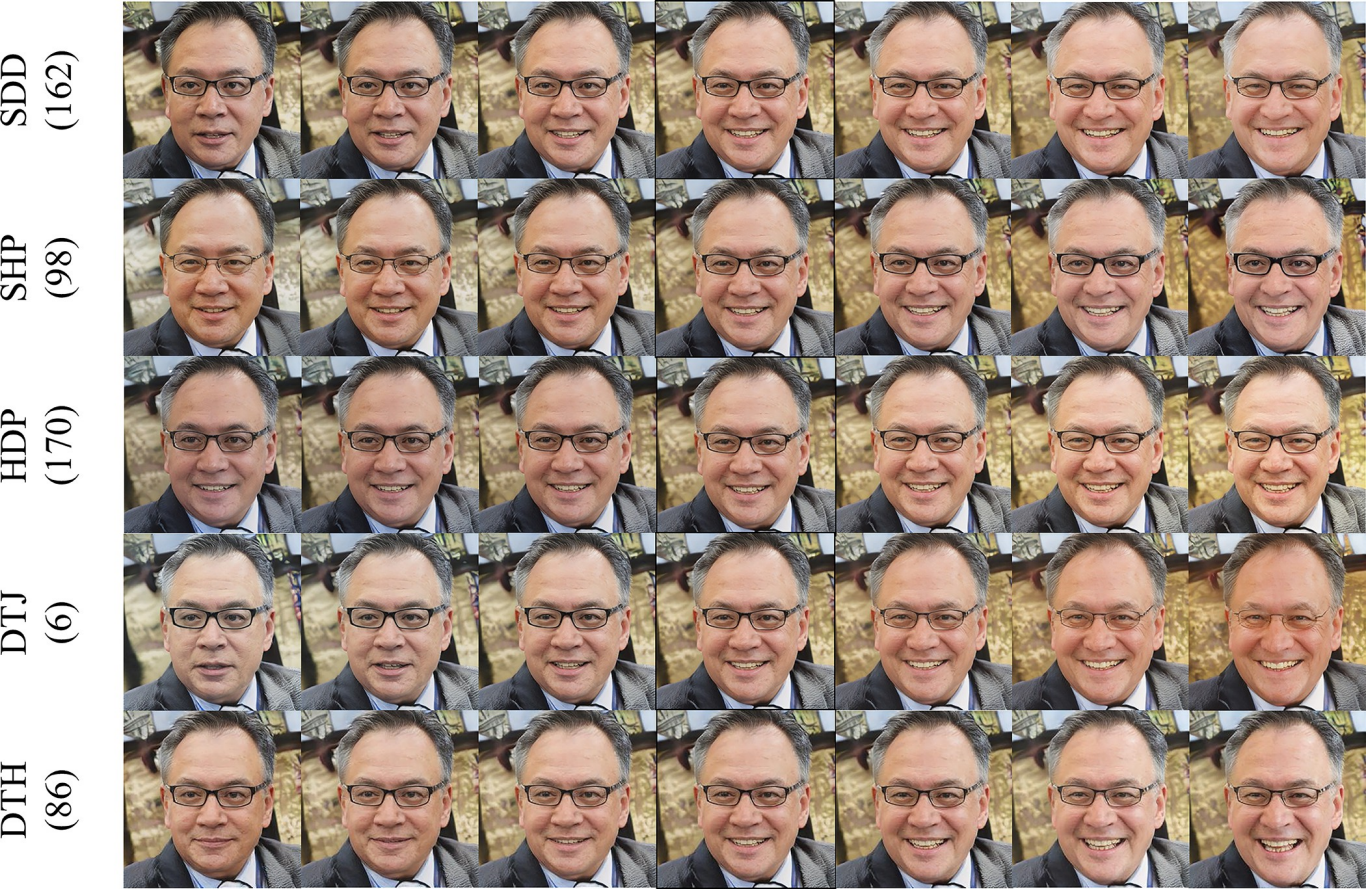
Editing results of different methods for the image attribute au_12_Lip_Corner_Puller.

Fig 3 shows the results of editing the image attribute au_12_Lip_Corner_Puller by various methods. If the size of the image is too small for detailed observation, please zoom in it for viewing or go to the source code website to view the original image and GIF animation.

Each row in Fig 3 represents a method. The number in brackets to the right of the method name is the index for the editing direction. The image with the border in the middle is the original image. The left side of the original image depicts the negative direction, and the right side denotes the positive direction. The SDD method is entangled with the age, skin color, lens color, and other attributes of the face while editing the attribute *au_12_Lip_Corner_Puller*. The SHP method is entangled with lens color, age, but it performs better than the SDD method to address the entanglement, but the same step duration modifies the *au_12_Lip_Corner_Puller* attribute to a smaller level. The application of the HDP method on *au_12_Lip_Corner_Puller* engenders minimal modifications as it is entangled with skin color, eyebrows, wrinkles and other attributes. As for the DTJ’s modification of the *au_12_Lip_Corner_Puller* attribute, it is the largest of the first four rows, but it is entangled with skin color, age, and seriously entangled with whether to wear glasses. Finally, the DTH method has the biggest edit magnitude for the corresponding attribute and is almost completely disentangled from other attributes. In comparison, our method works best for direction discovery and disentanglement.

**Fig 4:**
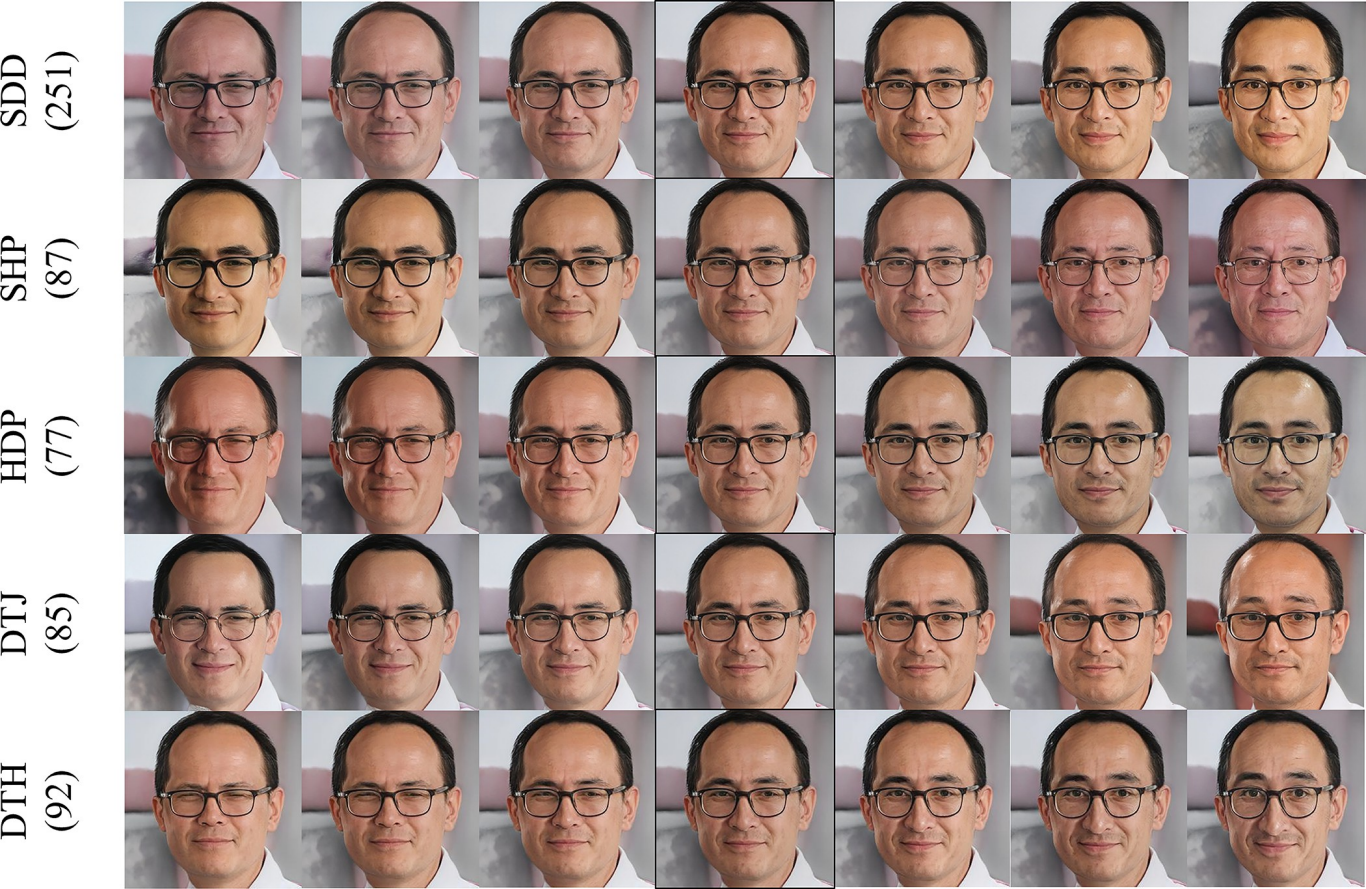
Editing results of different methods for the image attribute au_1_Inner_Brow_Raiser.

Fig 4 shows the editing results of various methods for the image attribute *au_1_Inner_Brow_Raiser*.

The method with the largest editing range for the attribute *au_1_Inner_Brow_Raiser* is DTH, and the method with the smallest editing range is HDP. The SDD method is entangled with skin color, hair, yaw, etc. whereas the SHP is entangled with age, skin color and the HDP is entangled with skin color, eyes, and beard. As for the DTJ method is entangled with hair.

By showing the editing effects in Figs 3 and 4, the HDP method has the smallest magnitude of editing for the relevant attributes, indicating that the editing direction discovered is not accurate. This occurrence is consistent with the analysis of the conflict between disentanglement learning and direction discovery in the training process presented in section 3.3 as it further demonstrates the need to use two-stage training. In terms of disentanglement, DTH and DTJ beat SDD and SHP methods, again demonstrating the need for disentangled learning in the second stage. Finally, SDD outperforms SHP in the editing magnitude of related attributes but has the largest number of other attributes coupled with it, indicating that the SDD method is more suitable for direction discovery while the SHP method is more suitable for disentanglement learning.

### Quantitative evaluation of disentanglement

Concerning the quantitative evaluation, a sequence of images is formed by taking the same number of steps along the positive and negative directions of the editing direction, and the attributes of each image are scored using the pre-trained network to obtain the scoring matrix *Score∈R*^*k×a×s*^, where *k* denotes the number of directions, *a* denotes the number of attributes, and *s* represents the length of the image sequence. The Pearson’s correlation was calculated for each attribute to get the correlation coefficient matrix *Corr∈R*^*k×a*^. The *n Corr* matrices are obtained by multiple sampling in the latent space and their average value is represented by *Corr*_*avg*_*∈R*^*k×a*^. The column attributes of *Corr*_*avg*_ are sorted in a descending order, and the semantics of the first ranked corresponding direction represents the attribute. An overview of the greatest correlation coefficients of some attributes is provided in Table 1A–1E.

**Table 1:** Comparison of Mean Pearson’s correlation for all methods.

** **	**gender**	**age**	**Brow_Raiser**	**Cheek_Raiser**	**Lip_Puller**	**Lips_part**
gender	**0.33**	0.12	0.04	0.04	0.01	0.03
age	0.03	**0.45**	0.11	0.05	0.18	0.02
Brow_Raiser	0.05	0.11	**0.52**	0.17	0.07	0.06
Cheek_Raiser	0.02	0.06	0.09	**0.45**	0.2	0.12
Lip_Puller	0.03	0.01	0.11	0.11	**0.55**	0.21
Lips_part	0.01	0.07	0.03	0.22	0.2	**0.51**
(a) Mean Pearson’s correlation for a subset of attributes in DTJ.						
	gender	age	Brow_Raiser	Cheek_Raiser	Lip_Puller	Lips_part
gender	**0.35**	0.11	0.16	0.13	0.07	0.05
age	0.03	**0.42**	0.19	0.08	0.16	0.01
Brow_Raiser	0.04	0.13	**0.46**	0.13	0.08	0.06
Cheek_Raiser	0.03	0.11	0.15	**0.39**	0.17	0.12
Lip_Puller	0.05	0.01	0.16	0.08	**0.43**	0.2
Lips_part	0.03	0.08	0.08	0.024	0.21	**0.45**
(b) Mean Pearson’s correlation for a subset of attributes in SDD.						
	gender	age	Brow_Raiser	Cheek_Raiser	Lip_Puller	Lips_part
gender	**0.3**	0.09	0.19	0.16	0.04	0.09
age	0.03	**0.34**	0.16	0.09	0.13	0.02
Brow_Raiser	0.02	0.19	**0.46**	0.22	0.1	0.14
Cheek_Raiser	0.03	0.2	0.13	**0.43**	0.28	0.16
Lip_Puller	0.03	0.02	0.03	0.25	**0.4**	0.31
Lips_part	0.01	0.12	0.05	0.24	0.33	**0.41**
(c) Mean Pearson’s correlation for a subset of attributes in SDD.						
	gender	age	Brow_Raiser	Cheek_Raiser	Lip_Puller	Lips_part
gender	**0.24**	0.07	0.02	0.01	0.11	0.03
age	0.02	**0.33**	0.14	0.06	0.13	0.18
Brow_Raiser	0.07	0.12	**0.4**	0.12	0.06	0.02
Cheek_Raiser	0.02	0.03	0	**0.37**	0.27	0.14
Lip_Puller	0.02	0.03	0.11	0.3	**0.34**	0.27
Lips_part	0.01	0.02	0.05	0.23	0.28	**0.32**
(d) Mean Pearson’s correlation for a subset of attributes in SHP.						
	gender	age	Brow_Raiser	Cheek_Raiser	Lip_Puller	Lips_part
gender	**0.24**	0.07	0.02	0.01	0.11	0.03
age	0.02	**0.33**	0.14	0.06	0.13	0.18
Brow_Raiser	0.07	0.12	**0.4**	0.12	0.06	0.02
Cheek_Raiser	0.02	0.03	0	**0.37**	0.27	0.14
Lip_Puller	0.02	0.03	0.11	0.3	**0.34**	0.27
Lips_part	0.01	0.02	0.05	0.23	0.28	**0.32**
(e) Mean Pearson’s correlation for a subset of attributes in HDP.						

The magnitude of the Pearson correlation coefficient represents the relationship between the score of the image attribute and the score index. Since the step size remains constant as one moves along the editing path, a correlation coefficient that is high indicates a larger change in the attribute. Table 1A displays that our method yields the maximum correlation coefficient of 0.55 for the attribute *au_12_Lip_Corner_Puller*. Observing Fig 3, we can see that our method has the greatest modification range for this attribute. Fig 4 and Table 1 also illustrate this phenomenon.

On the gender attribute, our method’s Mean Pearson’s correlation is lower than that of DTJ, and our method achieves the highest correlation coefficient for other attributes, whereas the majority of other methods achieve lesser correlation coefficients. This can be clearly visualized by the non-diagonal elements of each table. Thus, the results of the quantitative evaluation were consistent with the conclusions drawn from the qualitative comparison.

**Fig 5:**
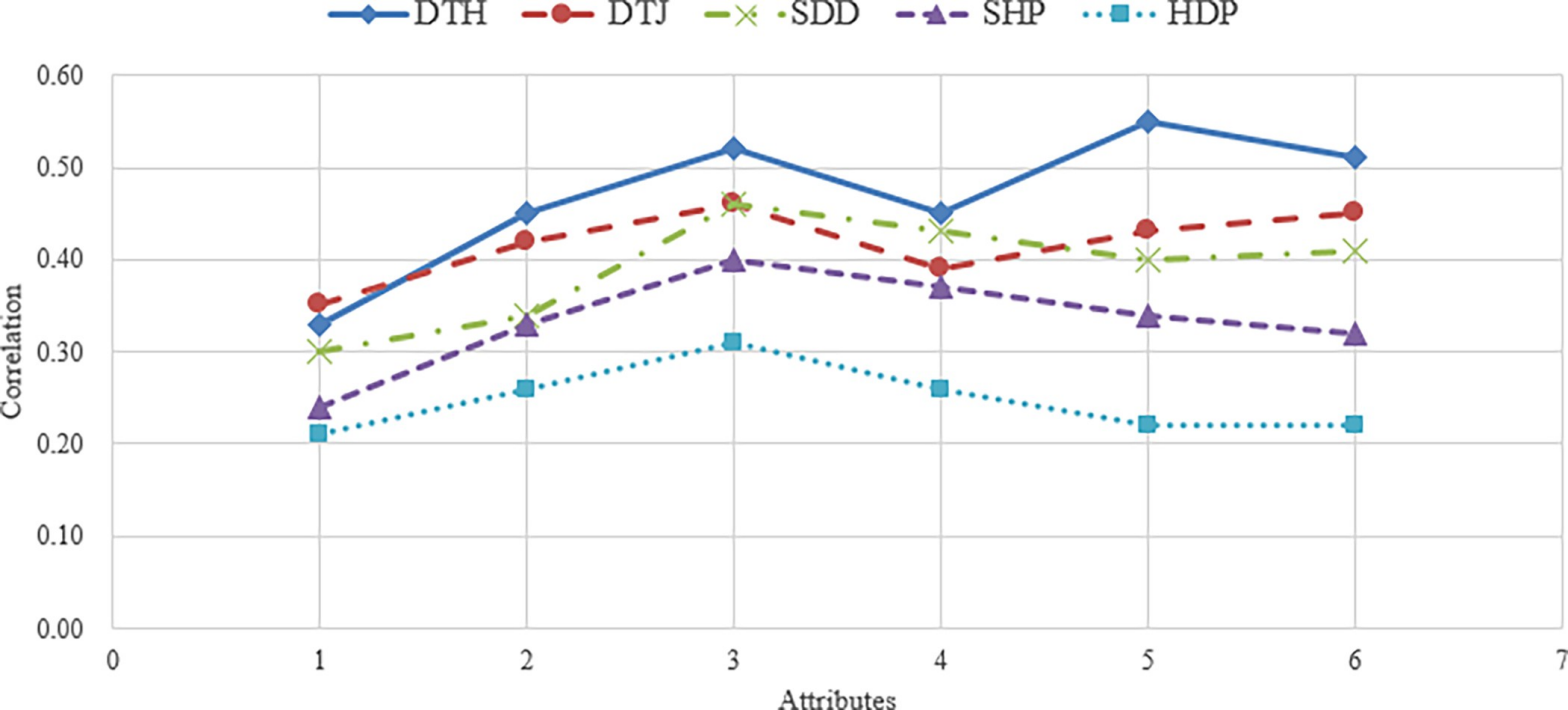
Comparison of correlation coefficients of attributes in all methods.

Fig 5 shows the comparison of the correlation coefficients for some attributes using all the methods, which can reflect more clearly the disentanglement performance of each method. The worst result is obtained by training the direction discovery and the disentanglement learning simultaneously, i.e., HDP. Although the non-diagonal elements of the matrix of the HDP method are small, the correlation coefficients for the corresponding attributes are also low, mainly because both components, i.e., disentanglement learning and direction discovery, conflict with each other throughout the training process. The experimental results suggest that our method has greater performance in terms of disentanglement and **Hessian Penalty is more suitable for disentanglement learning than for direction discovery**.

**Fig 6:**
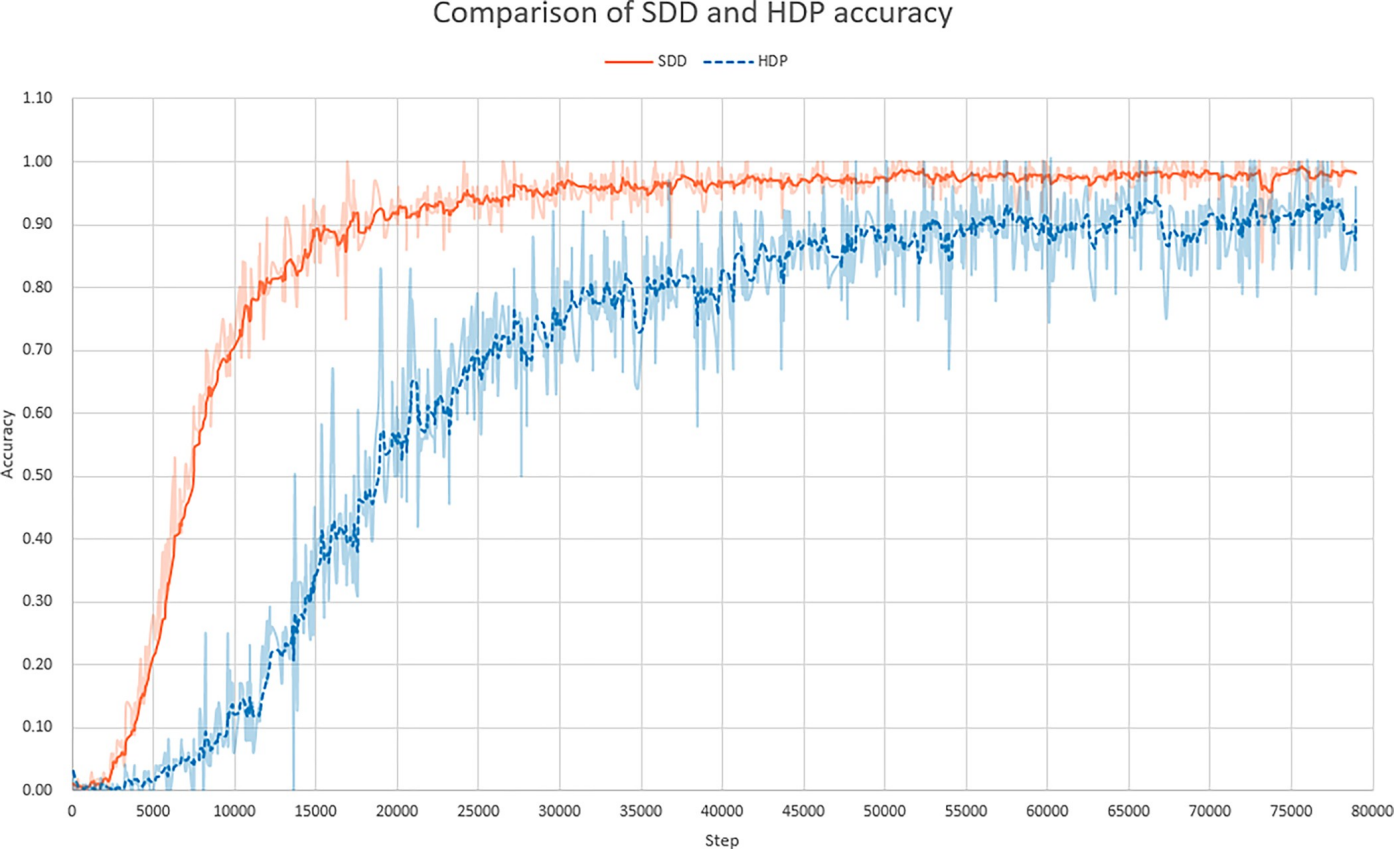
Comparison of the accuracy of two methods, SDD and HDP, in the training process.

As for Fig 6, it shows the comparison of the accuracy rate between the SDD and the HDP methods during the training process.

Observing Fig 6, the classification accuracy of the SDD method is significantly greater than that of the HDP method in the early training period, and the reason is that the Hessian Penalty and the discovery editing direction conflict with each other. In fact, the Hessian Penalty pulls down the accuracy of SDD method. The degree of oscillation of the two curves reveals that the Hessian Penalty interferes greatly with the directional discovery. The comparison of the accuracy rates verifies, once again, the analysis in Section 3.3 and provides a strong data support for the two-stage training.

## Conclusions

In this paper, we propose a two-stage training method for face editing. The method is unsupervised and model-agnostic, and it provides better performance in disentanglement by Hessian Penalty. After some theoretical research and experimental validation, it was demonstrated that the two-stage training generated the best desirable results for face editing in the StyleGAN2 model. Since the fundamental focus of the two-stage operation is the direction matrix placed outside the generative model, our method is independent of the specific generative model and can be easily adapted to other models, which was not the case of existent methods.

Finally, by calculating the mean Pearson’s correlation coefficients between the attribute scores of the image sequences and the indexes of the traversal paths, the disentanglement ability of the editing directions is quantitatively evaluated, and the ranking of the attribute correlation coefficients can automatically determine the semantics of the editing directions. Although the two-stage training method increases the training duration, the resulting editing model does not affect its application in other fields. However, the Hessian Penalty is a relatively weak method and does not achieve the desired result for the disentanglement of editing direction.

There are various factors that have an impact on direction discovery and direction disentanglement, such as the spatial structure of latent space. Currently, the research is based on the assumption that the editing direction is linear, and although some exciting results have been obtained, they are not very satisfactory, and future research on nonlinear editing directions may be required. There is a hierarchical structure to the majority of generative models, and the roles and semantics of the different layers are still unclear. It has been observed that the same editing direction operates differently in different layers, and a study of the semantics of the hierarchical structure of the generative models may assist in improving the editing of images. The search for stronger methods of disentanglement and new methods for simultaneous direction discovery and direction disentanglement in one stage are also the focus of future research.

## References

[1] Goodfellow I, Pouget-Abadie J, Mirza M, Xu B, Warde-Farley D, Ozair S, et al. Generative adversarial nets. In: Ghahramani Z, Welling M, Cortes C, Lawrence N, Weinberger KQ, editors. Advances in neural information processing systems. Curran Associates, Inc.; 2014.

[2] Brock A, Donahue J, Simonyan K. Large Scale GAN Training for High Fidelity Natural Image Synthesis. arXiv; 2019. doi: 10.48550/arXiv.1809.11096

[3] Karras T, Aila T, Laine S, Lehtinen J. Progressive Growing of GANs for Improved Quality, Stability, and Variation. 2018; 26. doi: 10.48550/arXiv.1710.10196

[4] Karras T, Laine S, Aila T. A Style-Based Generator Architecture for Generative Adversarial Networks. arXiv; 2019. doi: 10.48550/arXiv.1812.04948 32012000

[5] Karras T, Laine S, Aittala M, Hellsten J, Lehtinen J, Aila T. Analyzing and Improving the Image Quality of StyleGAN. 2020 IEEE/CVF Conference on Computer Vision and Pattern Recognition (CVPR). 2020 [cited 25 Sep 2021]. doi: 10.1109/cvpr42600.2020.00813

[6] Karras T, Aittala M, Hellsten J, Laine S, Lehtinen J, Aila T. Training Generative Adversarial Networks with Limited Data. arXiv:200606676 [cs, stat]. 2020 [cited 21 Sep 2021]. doi: 10.5555/3495724.3496739

[7] Shen Y, Gu J, Tang X, Zhou B. Interpreting the Latent Space of GANs for Semantic Face Editing. 2020 IEEE/CVF Conference on Computer Vision and Pattern Recognition (CVPR). Seattle, WA, USA: IEEE; 2020. pp. 9240–9249. doi: 10.1109/CVPR42600.2020.00926

[8] Goetschalckx L, Andonian A, Oliva A, Isola P. GANalyze: Toward Visual Definitions of Cognitive Image Properties. 2019 IEEE/CVF International Conference on Computer Vision (ICCV). Seoul, Korea (South): IEEE; 2019. pp. 5743–5752. doi: 10.1109/ICCV.2019.00584

[9] Spingarn-Eliezer N, Banner R, Michaeli T. GAN “Steerability” without optimization. arXiv; 2021. doi: 10.48550/arXiv.2012.05328

[10] Jahanian A, Chai L, Isola P. On the “steerability” of generative adversarial networks. 2020; 31. doi: 10.48550/arXiv.1907.07171

[11] Plumerault A, Borgne HL, Hudelot C. Controlling generative models with continuous factors of variations. arXiv; 2020. doi: 10.48550/arXiv.2001.10238

[12] Shen Y, Zhou B. Closed-Form Factorization of Latent Semantics in GANs. 2021 IEEE/CVF Conference on Computer Vision and Pattern Recognition (CVPR). Nashville, TN, USA: IEEE; 2021. pp. 1532–1540. doi: 10.1109/CVPR46437.2021.0015810.1109/CVPR46437.2021.00158

[13] Härkönen E, Hertzmann A, Lehtinen J, Paris S. GANSpace: Discovering Interpretable GAN Controls. arXiv. 2021. doi: 10.48550/arXiv.2004.02546

[14] Voynov A, Babenko A. Unsupervised Discovery of Interpretable Directions in the GAN Latent Space. Proceedings of the 37th International Conference on Machine Learning. PMLR; 2020. pp. 9786–9796. doi: 10.5555/3524938.3525845

[15] Peebles W, Peebles J, Zhu J-Y, Efros A, Torralba A. The Hessian Penalty: A Weak Prior for Unsupervised Disentanglement. Computer Vision–ECCV 2020. 2020; 581–597. doi: 10.1007/978-3-030-58539-6_35

[16] Zhu X, Xu C, Tao D. Learning Disentangled Representations with Latent Variation Predictability. arXiv; 2020. doi: 10.48550/arXiv.2007.12885v1

[17] Shen Y, Yang C, Tang X, Zhou B. InterFaceGAN: Interpreting the Disentangled Face Representation Learned by GANs. IEEE Trans Pattern Anal Mach Intell. 2022;44: 2004–2018. doi: 10.1109/TPAMI.2020.3034267 33108282

[18] Ding F, Fan B, Shen Z, Yu K, Srivastava G, Dev K, et al. Securing Facial Bioinformation by Eliminating Adversarial Perturbations. IEEE Trans Ind Inf. 2023;19: 6682–6691. doi: 10.1109/TII.2022.3201572

[19] Ding F, Zhu G, Li Y, Zhang X, Atrey PK, Lyu S. Anti-Forensics for Face Swapping Videos via Adversarial Training. IEEE Trans Multimedia. 2022;24: 3429–3441. doi: 10.1109/TMM.2021.3098422

[20] Dupont E. Learning Disentangled Joint Continuous and Discrete Representations. Advances in Neural Information Processing Systems. Curran Associates, Inc.; 2018. doi: 10.5555/3326943.3327009

[21] Miyato T, Kataoka T, Koyama M, Yoshida Y. Spectral Normalization for Generative Adversarial Networks. arXiv; 2018. doi: 10.48550/arXiv.1802.05957

[22] Jeong Y, Song HO. Learning Discrete and Continuous Factors of Data via Alternating Disentanglement. doi: 10.48550/arXiv.1905.09432

[23] Wei Y, Shi Y, Liu X, Ji Z, Gao Y, Wu Z, et al. Orthogonal Jacobian Regularization for Unsupervised Disentanglement in Image Generation. 2021 IEEE/CVF International Conference on Computer Vision (ICCV). 2021. doi: 10.1109/iccv48922.2021.00665

[24] Radford A, Metz L, Chintala S. Unsupervised Representation Learning with Deep Convolutional Generative Adversarial Networks. arXiv; 2016. doi: 10.48550/arXiv.1511.06434

[25] He K, Zhang X, Ren S, Sun J. Deep Residual Learning for Image Recognition. 2016 IEEE Conference on Computer Vision and Pattern Recognition (CVPR). Las Vegas, NV, USA: IEEE; 2016. pp. 770–778. doi: 10.1109/CVPR.2016.90

[26] Liu Z, Luo P, Wang X, Tang X. Deep Learning Face Attributes in the Wild. 2015 IEEE International Conference on Computer Vision (ICCV). 2015; 9. doi: 10.1109/ICCV.2015.425

[27] Wu Z, Lischinski D, Shechtman E. StyleSpace Analysis: Disentangled Controls for StyleGAN Image Generation. 2021 IEEE/CVF Conference on Computer Vision and Pattern Recognition (CVPR). Nashville, TN, USA: IEEE; 2021. pp. 12858–12867. doi: 10.1109/CVPR46437.2021.01267

[28] Cherepkov A, Voynov A, Babenko A. Navigating the GAN Parameter Space for Semantic Image Editing. 2021 IEEE/CVF Conference on Computer Vision and Pattern Recognition (CVPR). 2021; 10. doi: 10.1109/CVPR46437.2021.00367

[29] Wang B, Ponce CR. The Geometry of Deep Generative Image Models and its Applications. International Conference on Learning Representations. 2021. doi: 10.48550/arXiv.2101.06006

[30] Zhang R, Isola P, Efros AA, Shechtman E, Wang O. The Unreasonable Effectiveness of Deep Features as a Perceptual Metric. 2018 IEEE/CVF Conference on Computer Vision and Pattern Recognition. Salt Lake City, UT: IEEE; 2018. pp. 586–595. doi: 10.1109/CVPR.2018.00068

[31] Chen X, Duan Y, Houthooft R, Schulman J, Sutskever I, Abbeel P. InfoGAN: interpretable representation learning by information maximizing generative adversarial nets. Proceedings of the 30th International Conference on Neural Information Processing Systems. Red Hook, NY, USA: Curran Associates Inc.; 2016. pp. 2180–2188. doi: 10.5555/3157096.3157340

[32] Shoshan A, Bhonker N, Kviatkovsky I, Medioni G. GAN-Control: Explicitly Controllable GANs. 2021 IEEE/CVF International Conference on Computer Vision (ICCV). Montreal, QC, Canada: IEEE; 2021. pp. 14063–14073. doi: 10.1109/ICCV48922.2021.01382

[33] Zhang, S.; Zhu, X.; Lei, Z.; Shi, H.; Wang, X.; Li, S.Z. S3FD: Single Shot Scale-Invariant Face Detector. In Proceedings of the 2017 IEEE International Conference on Computer Vision (ICCV); IEEE: Venice, 2017; pp. 192–201.

[34] Kärkkäinen, K.; Joo, J. FairFace: Face Attribute Dataset for Balanced Race, Gender, and Age for Bias Measurement and Mitigation. In Proceedings of the 2021 IEEE Winter Conference on Applications of Computer Vision (WACV), 2021, pp. 1547–1557.

[35] Deng J.; Guo J.; Yang J.; Xue N.; Kotsia I.; Zafeiriou S. ArcFace: Additive Angular Margin Loss for Deep Face Recognition 2015. 14, 17. 10.1109/TPAMI.2021.3087709 34106845

[36] Mavadati S.M.; Mahoor M.H.; Bartlett K.; Trinh P.; Cohn J.F. DISFA: A Spontaneous Facial Action Intensity Database. IEEE Transactions on Affective Computing 2013, 4, 151–160.

[37] Mavadati, M.; Sanger, P.; Mahoor, M.H. Extended DISFA Dataset: Investigating Posed and Spontaneous Facial Expressions. In Proceedings of the 2016 IEEE Conference on Computer Vision and Pattern Recognition Workshops (CVPRW); IEEE: Las Vegas, NV, USA, 2016; pp. 1452–1459.

